# Alexander’s Operation

**Published:** 1887-01

**Authors:** Herman Mynter

**Affiliations:** Surgeon Buffalo General Hospital; Copenhagen


THE BUFFALO Medical AND Surgical Journal

VOL. XXVI. JANUARY, 1887. No. 6

### Original Communications.

### *ALEXANDER'S OPERATION.*

BY HERMAN MYNTER, M. D., Surgeon Buffalo General Hospital.

The Alexander operation, also called the Alexander-Adams operation (after Alexander in Liverpool and Adams in Glasgow) is still new, and rather without precise indications. It consists in shortening the round ligaments, and was proposed for retro-deviations and prolapse of the uterus by William Alexander in 1884. (The treatment of backward displacements of the uterus, and of prolapsus uteri by the new method of shortening the round ligaments, London, 1884). I have lately had an opportunity of seeing it performed by Prof. Studsgaard in the first surgical ward at the Commune Hospital, and as his case offers an unusual complication, I will, with his permission, add it to this short paper.

The operation, strictly speaking, is of French origin, as Tillaux says, the originator being M. Alguie, who shortened the ligaments in prolapse of the uterus to prevent the uterus from falling down, and Aran, who used it for retroflexion, believing that the role of the ligaments was less to suspend the uterus than to hold the fundus uteri forward.

Alexander's attention was, in 1879-81, attracted by the large number of cases of displacement of the uterus in the gynæcological ward of the Liverpool Workhouse. Prolapsus uteri was the most common, and next in order of frequency to retro deviations. Most of these cases from time to time, as it usually goes, had been treated with pessaries of different forms, but generally they could be tolerated only for a short time or not at all. The uterine complaint was the grand excuse to secure exemption from work and admission to the hospital. Alexander had formerly performed the usual operations on the vagina and perineum for prolapse, but was not satisfied with the results. He also had thought of stitching the uterus to the different parts of the pelvis, an operation which in the last number of *Centralblatt für Gynæcology* has been recommended by Olshausen, but at last his attention was called to the round ligaments, and, on Dec. 14, 1881, he operated successfully on a case of prolapsus uteri by pulling up and shortening the round ligaments. Since that time the operation has been performed a number of times, both in America and Europe, and I shall try, in this communication, to give your readers a short resumé of the different opinions in regard to its indications and contra-indications, technique and results. I have had the opportunity of performing the operation a number of times on dead bodies at the mortuary of the Commune Hospital.

*Anatomical Relations.*—According to Hule, the round ligaments (ligamenta teres uteri) take their origin from the upper angles of the uterus, one on either side, immediately in front of the Fallopian tube, proceed upwards and forwards towards the internal inguinal ring, pass through the inguinal canal and terminate, with most of their fibres, in the mons veneris and labia pudenda. A few of the fibres may be followed down to fascia pectinea and symphysis pubis. In the first part of its course, the ligament cannot be distinguished from the muscular fibres, which radiate out in the broad ligaments from the corpus uteri, but they soon gather together and form a cord five to seven millimeters thick, which, besides areolar tissue and vessels, contains plain muscular fibres like those of the uterus, from which they are prolonged. The ligaments are covered by the peritoneum in their whole length, being situated in a fold of the broad ligament. In the upper half of its course it is strongly united with the peritoneum, while the connection is very loose in the lower half. Sometimes in its course through the inguinal canal it is accompanied by a fold of peritoneum, a true processus vaginalis peritonei in the foetus, called the canal of Nuck. This canal is generally obliterated in the adult, but sometimes remains pervious to advanced age. Besides areolar tissue, vessels and organic muscular fibres, it contains whitish, shining fibrous filaments, the whole forming a semi-transparent, loose, brownish cord, as thick as a goose-quill. Some muscular fibres from the abdominal muscles, through which the inguinal canal passes, are added to the lower end, being an analogue to the cremaster muscles in the male. The part outside the inguinal canal consists mostly of connective tissue and fibrous filaments, is very delicate and apt to be destroyed by inexperienced operators, hence Alexander advises always to grasp the ligaments in the canal and never by the terminal fasciculi.

Alexander says, that in ordinary healthy individuals the round ligaments lie so loosely behind the peritoneum that that structure is scarcely disturbed by pulling the ligaments out, while in cases where pelvic cellulitis has taken place and adhesions have been formed, the round ligaments may fail to run, and they are found atrophied and brittle, caused by diminished vascular supply, from the constrictive influence of the cellulitis. The surgeon may, therefore, from the appearance of the ligament, judge whether it is judicious to continue the operation, as the ligaments in that case either will break in the canal, or rupture of the adherent peritoneum be produced. I have seen both things occur in operations on the dead, and that in cases where there was no signs of previous cellulitis. A large fold of the peritoneum is always drawn out with the ligament, the result of its strong connection with the upper half of the broad ligament, so large that in every case I have been able to pass a good-sized catheter through the inguinal canal from the abdominal cavity.

Alexander reports twenty-two cases operated by himself, and twenty-seven cases by other operators; eleven cases had prolapse of the uterus, eleven retro-deviations. The result was most satisfactory. In two of them but one ligament was shortened, but the result was just as good. In one case, which, on account of hystero-epilepsy, had had successively both arteriæ vertebrales ligated, the fits disappeared and the patient gave birth to a healthy child two years later. She had had no fits during those two years.

In one case, he removed the diseased left ovary through the wound, as it was easily pulled into the wound by the aid of the round ligament. In another similar case the same proceeding was tried but failed, the ovary being adherent. Alexander therefore prefers, for such cases, the usual oophorectomy, especially as much irritation is caused by this method, with considerable suppuration and slow healing of the wound.

Franzis Imlach, in Liverpool (see *Centralblatt für Gynæcology*, Nov. 17, 1886,) has made the operation thirty-six times; in nine cases on account of severe prolapse. Three of these nine cases were more than sixty years of age, with the result that one was perfectly relieved, another, who, before the operation, could not tolerate a pessary, was now able to wear a pessary, and felt well with its aid; the third, who had a large cystocele, continued to have trouble. Of the six younger patients, five were perfectly relieved, the sixth continued to complain of dysmenorrheal pains; twenty-seven cases were operated on account of retro-deviations (versions and flexions). In all those cases (16 out of 27), in which the deviation was not complicated with inflammatory conditions, the result was most satisfactory and the uterus continued to maintain the normal position. In only three cases there was no improvement, and these had chronic inflammation of the ovaries and tube. A number of cases, which, before the operation, continually miscarried, carried, after the operation, the embryo to full term.

William Polk, New York (see N. Y. *Medical Record*, July 3, 1886,) reports 15 cases. He is, thus far, satisfied with the results. One of his patients became pregnant later, and carried the child to full term without trouble. In another case he was compelled, from other reasons, to perform laparotomy ten months after the operation, and he found the uterus in good position.

Paul Munde' has reported six cases, two of which were successful, one partly successful; while he, in three cases, could not find the ligaments.

Dr. James Allan (the *Lancet*, June 7, 1884,) relates a case in which the operation was performed on account of prolapse of the uterus and mental disturbance at the monthly periods. Eighteen months after the operation the patient remained in good condition, the menstruation was normal, and she had no mental disturbance.

Sinclair also reports a successful case (see Edinburgh *Medical Journal*, September, 1885,) for prolapse of the uterus. The patient was 49 years of age; four months after the operation the uterus was anteverted, and a pessary was not necessary.

Halliday Croom reports, in the Edinburgh Obstetrical Association, a case in which an operation was performed on account of retroversion. It took him one hour to find one ligament, and he did not try to find the other. Nevertheless, the uterus continued in its new (normal) position, and the troubles were relieved.

Skene Keith has also operated once, and he joins Croom in the opinion that the operation is very difficult. Probably they both hunted after the terminal branches of the ligament, as he afterwards says that it is easy to find the inguinal canal and the ligament in it. His operation lasted one and a half hours. The uterus kept in anterversion for nine weeks, and then relapsed.

Leis (in Eufurt) has operated twice (see *Centralblatt für Gynæcology*, Nov. 29, 1886); once on account of subinvolution after miscarriage, in the fourth month. The result was good, although the uterus continued to be slightly prolapsed. The other case has not used any pessary for eight weeks, and the uterus continues in its normal position.

Rjasenzew (see discussion in St. Petersburg Obstetrical and Gynæcological Association) reports the result of seven cases, operated upon by Prof. Slavjansky. One case (prolapse, with retroversion,) was completely cured, as also were three cases of retroflexion. The operation was unsuccessful in two cases (one prolapse, and one retroflexion), and could not be finished in a third, for reasons not mentioned. As he considers the operation very difficult, the probability is, that he has not attempted to find the ligament in the inguinal canal, but has followed up the terminal fasciculi.

The case which has been operated on by Prof. Studsgaard, in Copenhagen, offers an unusual complication. The patient was an unmarried servant, 29 years of age, who, for about one year, had complained of difficulty when her bowels moved. The uterus was found strongly retroverted, but movable, and forming a strong prominence in the rectum, which increased when she bore down, and formed a kind of valve. Alexander's operation was performed on October 10, 1886, by aid of an incision acoss the mons veneris, from the ends of which an incision was made on both sides, in the direction of the canales inguinales. The round ligaments were found with greatest ease in the canals themselves, isolated and shortened about two inches, during which proceeding the left ligament broke, but the end of it was caught in the canal, and drawn out and sewed to the fascia with cat-gut. A fold of the peritoneum, which was drawn out with the ligament on the right side, was ligated, and cut off. The wound was closed with silk sutures, and a bandage of sublimatized cotton applied.

Oct. 11th—Retention of urine, which contains some blood; vomiting.

Oct. 13th—Severe pains in the abdomen.

Oct. 15th—Secretion of urine, through a fistula communicating with the incision on the right side; considerable secretion of pus from this side. A catheter was introduced into the bladder, and left in situ. She has no more trouble when her bowels move, and the uterus stands in slight anteversion.

Oct. 30th—She urinates voluntarily now, the fistula being almost closed.

Probably a part of the wall of the bladder was drawn out with the fold of the peritoneum, and enclosed in the ligature. I can see no advantage in the transverse incision made, although, as was expected, it healed by first intention.

*Technique.* —Alexander makes, after the bowels and bladder are emptied, and the pubes shaved, an incision, upwards and outwards from the pubic spine, in the direction of the inguinal canal, one or two inches in length, according to the amount of fat found. The external abdominal ring is now to be looked for, and, if not at once found, will be easily found by searching for the oblique fibres crossing it, and for a small quantity of fatty tissue issuing from its inner end. The landmark is the pubic spine, at which the outer or inferior pillar of the ring is inserted. Immediately inside this, the annular externus is found.

The oblique fibres crossing the external abdominal ring should next be cut across in the direction of the inguinal canal. A reddish tissue now bulges out, mixed with more or less fat. This is the end of the ligament—as a ligament just before it spreads out. An aneurism needle is now passed under all this fatty tissue, so as to raise it out of the canal and allow it to be grasped by the fingers. The ligament should now be gently pulled out, and all bands connecting it to the pillars of the ring or to the neighboring structures, should be cut through. The accompanying nerve, the genito-crural nerve, should also be cut through. In tearing the ligaments from their inguinal connections, some risk is run of breaking them, but when these adhesions are overcome, they pull with ease.

The uterus should now be placed in position with the sound, and the ligaments are then pulled out until they are felt to control the position of the uterus. Alexander warns against dragging the uterus into position by the ligaments. In backward displacement, he tries to put the uterus in a normal position; in prolapse, he pulls out the ligaments as far as they will come. They are then fixed to the pillars with two cat-gut sutures, passing through both pillars and the deepest part of the ligament; the wound is united with sutures, and bandaged.

A suitable Hodge pessary is to be introduced in the vagina, and the sound withdrawn. The patients are advised to lie well over on their side, the prone position being impossible to maintain. The wounds rarely heal by first intention, and the patients are kept in bed for twenty-one days. A couple of weeks later the pessary is removed, and the patient discharged.

In some aggravated cases of retroflexion, a stem pessary is necessary in addition to the Hodge pessary, for some time.

Imlach uses a very short incision (½ inch), which Alexander cautions against.

Gardner seeks for the fan-shaped distribution leading to the ligament, which is then caught in a forceps. The uterus having been replaced, per vaginam, the ligaments are drawn out 8–10 centimeters, and tied together over a tampon of Iodoform gauze. The ligaments are then fastened to the skin with cat-gut, a drainage tube introduced under the ligament, and the wound closed.

Performed in this way, the operation is very difficult; while, if the oblique fibres are cut through, and the ligament is caught in the inguinal canal, it is a very easy operation. The nerve, the genito-crural nerve, prevents the ligament from being drawn out unless it be cut through, and if this is neglected, and force be used in pulling, the ligament will break before the nerve does, which I have seen in a number of operations on the dead body.

*Indications and contra-indications.*—The operation has been criticised by many gynæcologists as unnecessary, as we have many other operative methods to relieve these troubles, but just this fact shows that there is room for improvement. It is not applicable to every case of retro-deviation or prolapse, as Alexander himself points out.

He considers the operation contraindicated in cases in which the uterus is so adherent that it cannot be replaced by the sound, and in cases in which pelvic cellulitis has once occurred, as the round ligaments cannot then be drawn out, even if the uterus can be replaced by the sound. But there are cases enough left where it may be done. According to Dr. Lohler, in Berlin, backward displacements of the womb form about one-fifth of the cases which come under the attention of the gynæcologist. Out of 240 such cases he had only four recoveries, by aid of pessaries, while Dr. Münde' recorded eight recoveries, by pessaries, out of 403 cases, and that, I believe, agrees with the experience of most physicians.

Some cases, as Alexander says, are completely relieved by pessaries, even if they are not cured, and here the operation is not indicated. About two per cent. are permanently cured by pessaries. In many the symptoms subside in the course of time, so that they can lay the pessary aside. But there are still many left who have no end of trouble from the use of pessaries; yes, in which they cannot be tolerated, who become nervous, hysterical, and are a continuous trouble to themselves and those around them, wandering from one doctor to another.

Just for such cases Alexander's operation is a boon, especially as it scarcely can be called a difficult one, if performed according to the rules he has laid down, and as there is scarcely any danger connected with it.

Imlach considers the operation chiefly indicated in those cases of retro-deviation in which the ovaries are dislocated, and, therefore, painful; while, if the primary trouble is found in the ovaries and tubes, the retro-deviation is a matter of secondary importance, and its correction would not benefit the original trouble, against which the treatment must then be directed.

Leis (in Eufurt) says, in *Centralblatt für Gynæcology*, Nov. 29, 1886, that the operation is not frequently performed in Germany. He considers it an objection, that, even after a successful operation, pessaries must be introduced to keep the portio vaginalis uteri backwards, and so give the weakened retro-uterine ligaments an opportunity to contract. We might truly say, that, if the operation only made it possible for patients to get along with the aid of pessaries, its performance was indicated in a number of cases, even if no cures were attained.

Winckel (in Munich) does not consider the results, so far, either particularly good or promising. The successful cases, he says, have not stood the test of time yet, and the anteversion produced is as much a deviation as the retro-deviation, and may, possibly, give as much trouble. The real cause of the retro-deviation, the weakened condition of the sacro-uterine ligaments, is not helped by the operation, and pessaries are as much necessary as before. He belives that the operation, in a short time, will become obsolete. He has the same opinion in regard to its performance for prolapse.

The objection to the anteversion produced is not correct, as it is not the intention to produce an anteversion in retro-deviations yet, as it is the fault of the operator who has shortened the ligaments too much. In regard to his objection to pessaries, the same may be said as previously, that much is gained if a patient is put in the position for wearing a pessary. Leis mentions a case in which the patient suffered from descensus uteri, retroflexion and lacerated cervix. Nothing helped her, neither bandages nor pessaries, nor Emmet's operation, and, as swelling and pains of the ovaries occurred, the operation was performed with the result that she was able to wear a pessary, and she felt well when the case was published, six weeks later. It is, to be sure, rather early to publish it, and the improvement may not continue.

While, in backward displacements, the sound ligaments should only be shortened sufficiently to keep the uterus in the normal position, Alexander advises, in prolapse, to shorten them as much as possible; produce anteversion of the womb. It is by all considered a *conditio sine qua non* that the perineum shall be intact, and by others, that it shall be a pure prolapse, uncomplicated by supra-vaginal elongation of cervix.

Lastly, it must be remembered that the operation can only cure diseases dependent on malposition and displacement of the uterus; if the uterus falls backwards again, then the operation is at fault, but if the uterus keeps in its new (normal) position, and the symptoms continue in spite of this, then the diagnosis is faulty, and the surgeon, not the operation, is to be blamed.

COPENHAGEN, November 10, 1886.

